# Translation initiation downstream from annotated start codons in human mRNAs coevolves with the Kozak context

**DOI:** 10.1101/gr.257352.119

**Published:** 2020-07

**Authors:** Maria S. Benitez-Cantos, Martina M. Yordanova, Patrick B.F. O'Connor, Alexander V. Zhdanov, Sergey I. Kovalchuk, Dmitri B. Papkovsky, Dmitry E. Andreev, Pavel V. Baranov

**Affiliations:** 1 School of Biochemistry and Cell Biology, University College Cork, Cork, T12 XF62 Ireland;; 2 Department of Computer Science and Artificial Intelligence, University of Granada, Granada, 18010 Spain;; 3 Shemyakin-Ovchinnikov Institute of Bioorganic Chemistry, RAS, Moscow, 117997 Russia;; 4 Belozersky Institute of Physico-Chemical Biology, Lomonosov Moscow State University, Moscow, 119992 Russia

## Abstract

Eukaryotic translation initiation involves preinitiation ribosomal complex 5′-to-3′ directional probing of mRNA for codons suitable for starting protein synthesis. The recognition of codons as starts depends on the codon identity and on its immediate nucleotide context known as Kozak context. When the context is weak (i.e., nonoptimal), leaky scanning takes place during which a fraction of ribosomes continues the mRNA probing. We explored the relationship between the context of AUG codons annotated as starts of protein-coding sequences and the next AUG codon occurrence. We found that AUG codons downstream from weak starts occur in the same frame more frequently than downstream from strong starts. We suggest that evolutionary selection on in-frame AUGs downstream from weak start codons is driven by the advantage of the reduction of wasteful out-of-frame product synthesis and also by the advantage of producing multiple proteoforms from certain mRNAs. We confirmed translation initiation downstream from weak start codons using ribosome profiling data. We also tested translation of alternative start codons in 10 specific human genes using reporter constructs. In all tested cases, initiation at downstream start codons was more productive than at the annotated ones. In most cases, optimization of Kozak context did not completely abolish downstream initiation, and in the specific example of *CMPK1* mRNA, the optimized start remained unproductive. Collectively, our work reveals previously uncharacterized forces shaping the evolution of protein-coding genes and points to the plurality of translation initiation and the existence of sequence features influencing start codon selection, other than Kozak context.

The initiation of protein synthesis during translation of most eukaryotic mRNAs typically occurs at an AUG start codon. The start codon is recognized by the preinitiation complex (PIC) consisting of a 40S ribosomal subunit, a Met-tRNA_i_^Met^, and several eukaryotic initiation factors (eIFs). The PIC scans the 5′ untranslated region (leader) in the 5′-to-3′ direction; and once a suitable codon is recognized as a start codon, some initiation factors dissociate, the 60S subunit joining occurs, and the process of protein synthesis ensues (for reviews, see Jackson et al. 2010; Hinnebusch 2014; Merrick and Pavitt 2018; Shirokikh and Preiss 2018). The recognition of initiation codons is stochastic: Not all PICs initiate at the first AUG codons, and some PICs continue scanning in the process termed leaky scanning (for review, see Kozak 1999). A recent single-molecule imaging study (Boersma et al. 2019) suggests that differences in conformations of individual mRNA molecules could be largely responsible for the observed heterogeneity in start codon selection. Nonetheless, on average the probability of PICs initiating at AUGs relies on their sequence context. Kozak reported that the 6 nt preceding the initiation codon and the nucleotide immediately downstream influence the translation initiation efficiency, with GCCRCCAUGG (R = A or G) being the consensus sequence in vertebrate mRNAs (Kozak 1987a,b). Specifically, −3 and +4 positions (+1 refers to A in AUG) were found to have a strong effect on translation initiation efficiency with A/G in −3 and G in +4 being optimal (Kozak 1986, 1997). There is a broad range of translation initiation efficiency values. These values for essentially all possible contexts (−6 to +5) have been recently characterized quantitatively with FACS-seq technique and RYMRMVAUGGC (Y = C or U; M = A or C; V = G, C or A) was found to have the strongest context supporting Kozak's initial results (Noderer et al. 2014).

Not surprisingly, the context of most AUGs that serve as start codons (sAUG) is close to the optimal owing to evolutionary selection to optimize translation efficiency of most mRNAs. However, there are many sAUGs in a weak context, and in certain cases, such weak context is preserved during evolution (Miyasaka et al. 2010; Loughran et al. 2018). The *EIF1* mRNA is a particularly striking example because its product influences context recognition and reduces initiation at AUGs in poor context (Martin-Marcos et al. 2011; Fijalkowska et al. 2017). Thus, the poor context of *EIF1* sAUG serves as a sensor and a modulator of eIF1 levels as the initiation rate increases when eIF1 levels drop and vice versa (Ivanov et al. 2010).

When sAUG is in a poor context, a proportion of ribosomes do not recognize it and continue scanning and may initiate at the next first downstream AUG (fdAUG). When fdAUG occurs in the same frame (in-frame) with sAUG, the synthesis of a truncated proteoform would be expected (Fig. 1A, top). Such truncated proteoforms may retain full or partial activity of the full protein product; for example, the N terminus could encode a particular localization signal and alternative products could be sent to different cellular compartments or secreted. Ribosome profiling combined with N-terminal proteomics revealed the existence of many such proteoforms (Menschaert et al. 2013; Van Damme et al. 2014). When expression of alternative proteoforms is evolutionarily advantageous, we may expect alternative initiation starts to be conserved; indeed it has been shown that in-frame start codons are more conserved downstream from sAUGs in the weak context in comparison with sAUGs in the strong context (Bazykin and Kochetov 2011).

**Figure 1.**
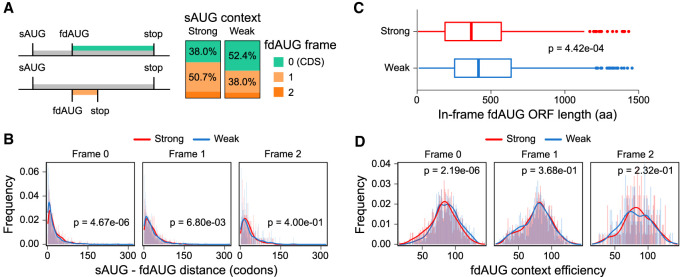
Relationship between Kozak context of sAUGs and locations of fdAUGs. (*A*) A schematic for classification of fdAUGs as in-frame and out-of-frame is shown on the *left*. Bar plots on the *right* show the proportion of fdAUGs in three different frames relative to sAUGs. (*B*) Distribution of distances between sAUGs and fdAUGs depending on fdAUG frame in strong and weak data sets (Mann–Whitney *U* test). (*C*) Boxplots showing distribution of lengths between in-frame fdAUGs and the next in-frame stop codons in the weak and strong sAUG data sets (Mann–Whitney *U* test). (*D*) The distribution of fdAUGs Kozak context strengths (as measured by Noderer et al. 2014) depending on fdAUG frame for strong and weak data sets (Student's *t*-test).

When fdAUG and sAUG are in different frames (out-of-frame), the initiation of a completely different protein would ensue (Fig. 1A, bottom). Such translation of the same mRNA fragments in more than one frame has indeed been observed in a small number of transcripts (Michel et al. 2012). Chung et al. (2007) carried out analysis of long (>500 nt) overlaps between ORFs and identified ∼150 cases that are conserved between human and mouse (∼1% of the tested orthologs). This number is significantly higher than what would be expected by chance (∼0.1%) if evolutionary selection acted only on a single frame. This strongly suggests that many such cases encode functional proteins in overlapping regions. The investigators conservatively estimated 40 among the ∼15,000 tested orthologs (Chung et al. 2007). Regardless of the true value, it seems that the great majority of protein-coding ORFs do not encode proteins in alternative reading frames. This is also evident from the difference in substitution rates of synonymous and nonsynonymous codons (Kimura 1977). Thus, in most cases, translation of an alternative reading frame is unlikely to produce a useful molecule. It would be wasteful and perhaps even harmful, with rare exceptions leading to de novo emerging functional peptides. If so, globally, we would expect an evolutionary selection to prefer in-frame fdAUGs over out-of-frame fdAUGs. Therefore we reasoned that there should be an evolutionary relationship between the translation initiation context of the first AUG and the occurrence of downstream AUG codons. Here, we explored this relationship using phylogenetic approaches, available ribosome profiling, and mass spectrometry data. We also experimentally tested a few selected cases with predicted multiple translation initiation starts.

## Results

### The frequency of in-frame fdAUG codons is increased downstream from sAUGs in weak contexts

To test the dependence between initiation context of sAUGs and position and framing of fdAUG, we designed two data sets of human mRNAs with the most and the least efficient Kozak contexts. This was done by selecting transcripts with top 10% and bottom 10% sAUG contexts according to previously reported experimental measurements (Methods; Supplemental Tables S1, S2; Noderer et al. 2014), to which we further refer to as “strong” and “weak” sAUGs. Assuming that strong sAUGs are highly efficient and nonleaky, PIC should not pass downstream and therefore fdAUG codons are unlikely to function as starts. On the contrary, weak sAUGs are likely to be leaky and some PICs will proceed downstream and initiate at fdAUG. As previously outlined, we hypothesized that on most mRNAs, the evolutionary selection should prefer in-frame downstream initiation over out-of-frame initiation. Therefore, in-frame fdAUGs should be more frequent in the weak data set than in the strong data set. To test this hypothesis, we classified mRNAs based on the frame in which fdAUGs occur (Fig. 1A). It appears that in both data sets, fdAUGs are the least frequent in Frame 2, 11.3% in the strong and 9.6% in the weak data set. This is expected owing to avoidance of stop codons in Frame 0 because A downstream from AUG in Frame 2 would create UGA codon in Frame 0. The most frequent fdAUG frame in the weak data set is indeed Frame 0 (52.4.%), which is in stark contrast to the strong data set in which fdAUGs in Frame 0 occur only in 38.0% of mRNAs (χ^2^ test, *P* = 3.1 × 10^−10^).

We further argued that there could be an evolutionary selection for AUG codons between sAUGs in weak context and the next out-of-frame AUG. Specifically, a mutation leading to emergence of an in-frame AUG codon downstream from a weak sAUG but upstream of out-of-frame fdAUG would result in preventing the harmful effect of translation initiation at the out-of-frame fdAUG via recruiting leaked ribosomes to synthesize slightly shorter proteoforms of the main mRNAs protein products. To test this hypothesis, we measured the distances between sAUGs and fdAUGs in different frames (Fig. 1B). Indeed, it appears that the distance between sAUG and in-frame fdAUG is shorter in the weak data set than in the strong data set (Mann–Whitney *U* test, *P* = 3.76 × 10^−6^). The difference between the two data sets is nonsignificant when fdAUG is out-of-frame. Consistent with the above, the distance between in-frame fdAUG and the stop codon is also larger in the weak data set than in the strong data set (Fig. 1C).

If production of proteoforms with alternative N termini is evolutionary selected, intuitively the architecture of such mRNAs should contain a start codon in a weak context followed by a start codon in a strong context. In such mRNAs, fdAUG would be expected to be in a stronger context than fdAUG of coding sequences (CDS) encoding a single proteoform. To explore whether this is the case we analyzed the strength of in-frame and out-of-frame fdAUG contexts in strong and weak data sets (Fig. 1D). Indeed, in-frame fdAUGs appeared to have stronger context in the weak data set than in the strong data sets (Student's *t*-test; *P* = 3.38 × 10^−7^), whereas no such dependency was observed for out-of-frame fdAUGs.

### Strong fdAUG context is under evolutionary selection downstream from conserved weak sAUGs

**Figure 2.**
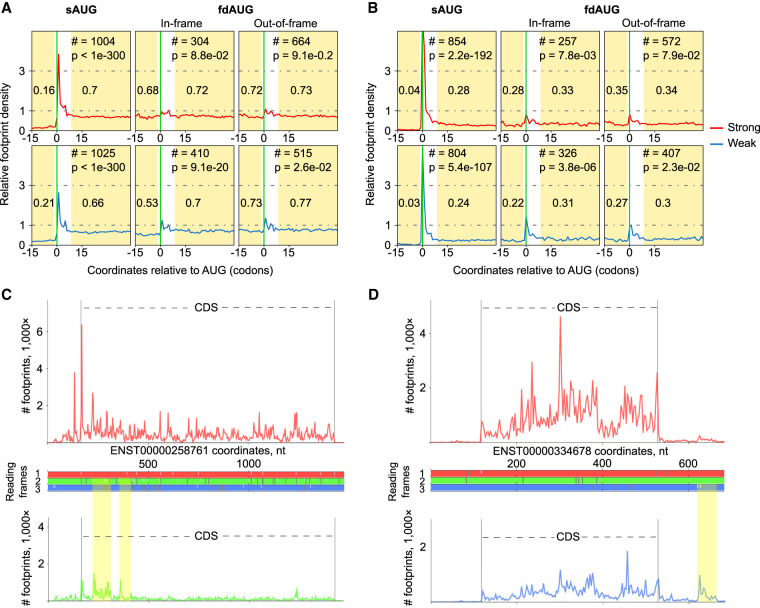
Analysis of ribosome profiling densities. (*A*) Relative ribosome footprint density (1 corresponds to average CDS density) surrounding sAUGs and fdAUGs. The areas used for calculating footprint density are highlighted, and the median density is indicated. The number of transcripts (#) used for generating metagene profiles as well as *P*-values as a measure of statistical significance (Mann–Whitney *U* test) are indicated at the *top*. (*B*) Same as *A* but for ribosome profiling data obtained with the methods that enrich footprints from initiating ribosomes. In this case, the size of the peak at AUG is more informative. (*C*) Subcodon ribosome footprint profiles for a *BZW2* transcript variant. (*Top*) Density profile of footprints supporting translation of CDS Frame 1; (*bottom*) density profile of footprints supporting translation of alternative reading frame; (*middle*) ORF plot with white dashes indicating AUG codons and gray dashes indicating stop codons. The colors used to indicate reading frames match subcodon profiles supporting translation of corresponding frames. Nested ORFs whose translation is supported by ribosome profiling data are highlighted in yellow. (*D*) Subcodon profile of *RPS19BP1* transcript variant. In this case, fdAUG and corresponding ORF whose translation is supported with ribosome profiling data occurs downstream from the CDS.

Although we found that in-frame fdAUGs are selected for downstream from weak sAUGs in comparison with strong sAUGs, nearly half of fdAUGs are out-of-frame (Fig. 1A). To assess translation initiation at fdAUG, we analyzed publicly available ribosome profiling data (Fig. 2). It shows metagene profiles for the footprint density surrounding in-frame and out-of-frame fdAUGs for ribosome profiling data based on capturing elongating (Fig. 2A) and initiating ribosomes (Fig. 2B). It can be seen that the change in ribosome profiling density occurring at fdAUGs is higher downstream from weak sAUGs (the changes downstream from strong sAUG are nonsignificant). The change is more clearly observed for in-frame fdAUGs downstream from weak sAUGs, and a less significant change is observed for out-of-frame fdAUGs. Figure 2C shows an example of nested ORFs translation from out-of-frame AUGs occurring downstream from *BZW2* sAUG that is known to have evolutionarily conserved weak context (Loughran et al. 2018). Moreover, translation at fdAUG can be observed in those cases in which fdAUG is located downstream from CDS in the 3′ UTR as in the example of *RPS19BP1* (Fig. 2D). Although it is possible that some nested or downstream ORFs code for functional peptides, this is unlikely to be the case for the majority of mRNAs with weak sAUGs because most nested ORFs initiated at fdAUG are very short (Supplemental Fig. S1), and mRNAs where fdAUG is downstream from CDS are rare (we found 76 examples in the entire catalog of principal isoforms). Also, we did not find a significant difference in the length of ORFs under control of out-of-frame fdAUGs between the weak and the strong data sets (Mann–Whitney *U* test, *P* = 1.99 × 10^−1^). Thus, the presence of a weak sAUG does not necessarily indicate that initiation downstream is evolutionary advantageous. In the case of in-frame fdAUGs, two proteoforms could differ just by a few amino acids at the N terminus and may be functionally indistinguishable, especially when the N terminus is proteolytically cleaved post-translationally. Nonetheless, if a significant proportion of mRNAs with weak sAUGs is responsible for the synthesis of alternative proteoforms with distinct functions or cellular localization and this increases the fitness, then we could expect that the AUG contexts (weak at sAUG and strong at fdAUG) would be under evolutionary selection.

To explore this, we analyzed the conservation of the contexts at sAUG and fdAUGs. Because the purpose was to focus on the context strength rather than nucleotide sequence, we devised the Translation Initiation Efficiency Selection Score (TIESS) (Methods, equation [1]). TIESS measures consistency with which sAUG contexts for orthologs in UCSC 100-way vertebrate alignment (Rosenbloom et al. 2015) are either stronger or weaker than average Kozak context across all sAUGs in the alignment. The context strength was calculated using experimental values obtained with FACS-seq for all possible contexts from −6 to +5 (Noderer et al. 2014). Therefore, the TIESS is designed to be the furthest (highest or lowest) for the conserved genes whose sAUG context consistently deviates the most from the average. TIESS would be close to zero for those cases in which the contexts are close to average or its deviation is not consistent (i.e., low in some species but high in others).

**Figure 3.**
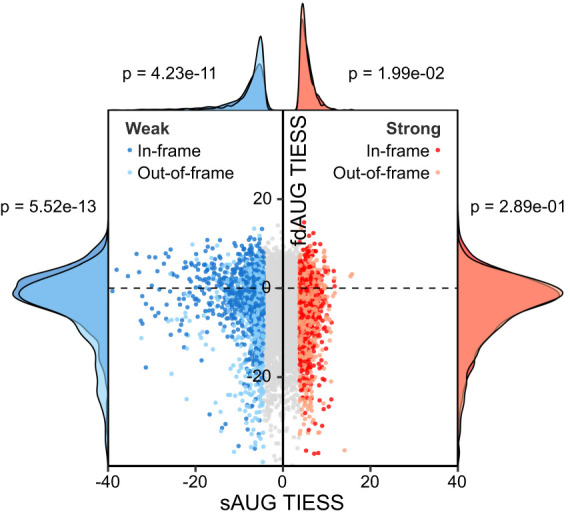
Relationship between evolutionary conservation of sAUG and fdAUG contexts. TIESS scores for conserved strong and weak sAUG codons and corresponding fdAUG codons. Although distribution of TIESS is indistinguishable for strong sAUGs and their fdAUGs (red histograms), contexts of weak sAUG with in-frame fdAUGs are more conserved than weak sAUG with out-of-frame fdAUGs (*top* blue histograms). At the same time there are more conservatively weak contexts for out-of-frame fdAUGs than for in-frame fdAUGs among mRNAs with weak sAUGs (*left* histograms).

After the translation efficiency conservation analysis, two new sets of transcripts were created with the 10% of the mRNAs with the highest (“conserved strong” set) (Supplemental Table S3) and the lowest (“conserved weak” set) (Supplemental Table S4) sAUG TIESS. We then analyzed the distribution of TIESS for both sAUGs and in-frame and out-of-frame fdAUGs (Fig. 3). First, conservation of weak sAUGs is stronger in mRNAs with in-frame fdAUGs (Mann–Whitney *U* test, *P* = 4.23 × 10^−11^). No significant difference was observed for the conservation of strong sAUGs depending on fdAUG frame. This indicates that evolutionary selection acts on the context of weak sAUGs in a proportion of mRNAs with in-frame fdAUGs, suggesting that the initiation at some fdAUGs is functionally significant. Further, in genes with weak sAUG context, out-of-frame fdAUGs are more frequently “conserved weak” than in-frame fdAUGs (Mann–Whitney *U* test, *P* = 5.52 × 10^−13^). Again, no such frame dependency is observed for fdAUG contexts from the conserved strong sAUG data set. This suggests that there is evolutionary selection acting to weaken context of out-of-frame fdAUGs in mRNAs with weak sAUG. This is likely attributable to potentially harmful effect of such out-of-frame initiation.

### Verification of downstream initiation at mRNAs containing sAUGs in evolutionarily conserved weak context using reporter constructs

**Figure 4.**
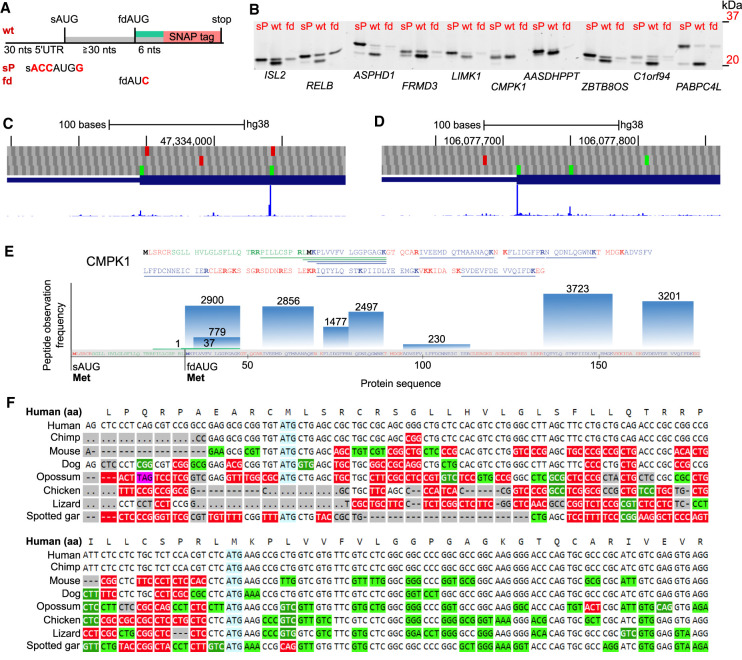
Experimental verification of downstream initiation at mRNAs containing sAUGs in evolutionarily conserved weak context. (*A*) Schematic of the test sequence cassette fused to SNAP tag. The wt test sequence includes 30 nt upstream of sAUG, the spacer between sAUG and fdAUG, and 6 nt downstream from fdAUG. For each wt test sequence there are two controls, sP has the sAUG in perfect Kozak context, and fd has the fdAUG changed to AUC. wt, sP, and fd test cassettes were designed for 10 selected genes. (*B*) Scans of protein gels used to separate SNAP-tagged protein products from test constructs expressed in HEK293T cells. Gene names are shown *below* the lanes. (*C*,*D*) GWIPS-viz screenshots for *CMPK1* and *AASDHPPT* loci, respectively. The *top* plots show codons in three reading frames with AUGs colored green and stop codons colored red. *Below* is RefSeq annotation of corresponding transcripts, and the thicker area of bars represents CDS. GWIPS-viz aggregated initiating ribosomes track is at the *bottom*, showing the density of footprints obtained from the ribosomes enriched at initiating sites with specific drugs. (*E*) Peptide observation frequency from PeptideAtlas for CMPK1 protein. In red are tryptic peptides not expected to be detectable. In green are detectable peptides unique for the long proteoform, and in blue are shared peptides. (*F*) Codon alignment of representative mammalian genomic sequences in the vicinity of sAUG and fdAUG (highlighted in blue) of *CMPK1* locus. Synonymous substitutions are highlighted in light green; nonsynonymous are shown in white font highlighted in green for similar and in red for dissimilar amino acids according to BLOSUM62.

To validate translation initiation at fdAUGs and to assess their efficiencies in specific human mRNAs, we designed a reporter based on SNAP tag, which allows visualization of reporter-containing proteins directly in the gel (Methods). This allows easy discrimination of protein products depending on their size. The schematic of the test construct is shown in Figure 4A. To test translation initiation at alternative starts, we manually selected 10 examples among the genes with the most conserved weak sAUGs. To facilitate visualization, we required at least 10 codons distance between sAUG and fdAUG. In addition, we avoided the presence of AUGs within 30 nt upstream of sAUG because of their likely inhibitory effect on translation initiation. This yielded transcripts of 10 human test genes: *ISL2*, *RELB*, *ASPHD1*, *FRMD3*, *LIMK1*, *CMPK1*, *AASDHPPT, ZBTB8OS*, *C1orf94*, *PABPC4L*. We chose not to introduce the entire 5′ leaders of the corresponding mRNAs because the annotated 5′ ends of RefSeq mRNAs may not reflect the most common starts of transcription and may even be heterogeneous (Gandin et al. 2016). For each of these, a test sequence cassette from 30 nt upstream of sAUG to 6 nt downstream from fdAUG, was fused upstream of SNAP tag encoding sequence with its AUG removed. In addition, two controls were designed for each case. In one control, sAUG context was replaced with the perfect Kozak context (denoted sP in Fig. 4A,B), and in the other control, fdAUG was replaced with AUC (denoted fd in Fig. 4A,B). Following transfection of cultured cells with the test plasmids, the cells were lysed in the presence of a SNAP tag substrate that binds SNAP covalently (Methods). Lysates were then separated on protein gels shown in Figure 4B for HEK293T cells and in Supplemental Figure S2 for HeLa and HEK293A, which have an additional series of constructs in which fdAUGs were placed in the perfect Kozak context (fdP in Supplemental Fig. S2).

Several observations could be made based on this analysis. First, in all of the tested mRNAs, the fdAUG supported higher translation initiation than sAUG with the most salient example of *CMPK1*, where initiation at the sAUG is below the level of detection. Another noteworthy case is *C1orf94*, in which, in addition to the expected products of sAUG and fdAUG initiation, there is an additional product that corresponds to initiation at a CUG codon located between the two AUGs (we confirmed this by substituting CUG with CUU) (denoted as cuu in Supplemental Fig. S2). Although CUG initiation downstream from AUG is highly unusual, it is not unprecedented: An example of functionally distinct proteoform initiated from such CUG has been reported for *MRPL18* (Zhang et al. 2015).

Unexpectedly, placing sAUG codons in an optimal context did not prevent initiation at fdAUGs for all but *ISL2* and *LIMK1* cases. The utmost case in this regard is *CMPK1,* in which initiation at fdAUG remained predominant even when sAUG was placed in an optimal context. Collectively these observations suggest that factors other than the immediate nucleotide context of an AUG can modulate the probability of translation initiation at particular starts. Even more so, the case of *CMPK1* suggests that such other factors can override the AUG context, and that initiation efficiency can be very low even when the immediate nucleotide context is optimal. We noticed the very GC-rich 5′ leader sequence of *CMPK1*, which may be involved in formation of a secondary structure or other interactions that somehow interfere with sAUG recognition while allowing initiation at fdAUG.

Another unexpected observation is that in some cases (i.e., *ZBTB8OS*, *ISL2*, and *C1orf94*) the intensity of the band corresponding to sAUG products is reduced when fdAUG is mutated to AUC consistently across all tested cell lines. One explanation could be that the ribosome initiating at fdAUG creates a roadblock and a ribosome queue that slows down PIC at sAUG increasing the chance that it will be recognized as a start. Such a mechanism has been proposed in plants (Dinesh-Kumar and Miller 1993). A similar mechanism has been shown recently to operate in certain mammalian non-AUG uORFs where initiation is induced by the ribosomes paused downstream (Ivanov et al. 2018).

Placing fdAUG in perfect Kozak context leads to only a marginal increase in intensity of bands corresponding to fdAUGs in some of the cases (Supplemental Fig. S2). This suggests that the native context of most fdAUGs is sufficient to capture most PICs leaked through sAUGs.

### Analysis of publicly available data supporting expression of alternative proteoforms

The relative efficiencies of translation initiation at alternative starts that can be estimated from ribosome profiling data available at GWIPS-viz (Michel et al. 2018) are consistent with some but not all of the tested examples. For example, the relative densities of initiating ribosomes at *CMPK1* sAUG and fdAUGs are consistent with our reporter assays (Fig. 4C), whereas for *AASDHPPT*, ribosome profiling data suggest that initiation at sAUG is greater than at fdAUG, contrary to our reporter assay (Fig. 4D). For intact GWIPS-viz screenshots of each of the 10 genomic loci, see Supplemental Figures S3–S12. A certain level of disagreement between the two approaches is to be expected, because only a part of the 5′ leader and of CDS was included in the constructs used here; thus, the reporters may not accurately reflect endogenous translation of full-length mRNAs. Furthermore, we tested these constructs in a limited number of cultured cell lines, and the regulation of translation initiation on these mRNAs may differ in cells from the tissues where these genes are normally expressed. In addition, the expression levels of reporter constructs differ from that of endogenous mRNAs, and this may also lead to differences. Although ribosome profiling has no such limitations, it is subject to several artifacts (Hussmann et al. 2015; O'Connor et al. 2016; Gerashchenko and Gladyshev 2017; Halpin et al. 2020) that may cause distortions between the real distribution of the ribosomes and the observed distribution of their footprints obtained with this technique. We also cannot exclude the possibility that the differences between the two approaches reflect the real biological difference between cell lines in which the experiments were executed.

We also sought support for the existence of alternative proteoforms using proteomics data. If two proteoforms are expressed from the same mRNA simultaneously, we would expect that among independent proteomics experiments the frequency of observations of tryptic peptides matching the sequence shared by these proteoforms will exceed the observation frequency of peptides that are unique to a single proteoform. We took peptide observation frequencies from PeptideAtlas (Desiere et al. 2006), which contains collective data from thousands of independent proteomics experiments for many hundreds of cell lines and tissue types. Figure 4E shows the peptide observation frequency distribution for *CMPK1*, and Supplemental Figures S13–S19 show the data for seven of nine remaining cases. Two cases were excluded. *C1orf94* has a very low expression and contains very few matching peptides, whereas the *PABPC4L* protein product sequence in PeptideAtlas is based on UniProt (The UniProt Consortium 2015), which contains only the truncated proteoform (accession ID P0CB38). In accordance with the reporter assay and ribosome profiling data, the peptides unique for the longer sAUG-starting proteoform are either missing or have hundred times lower observation frequency than the shared peptides, with the only exception of RELB protein (Supplemental Fig. S18). Within the shared protein sequence, all the expected tryptic peptides are consistently identified, and the only missing peptides belong to the regions unique for the longer proteoforms. At the same time, in addition to the start codon positioning, alternative explanations for the missing peptides are possible, for example, post-translational N-terminal peptidase cleavage.

For the unusual case of *CMPK1*, we also explored the conservation pattern using the CodAlignView tool (https://data.broadinstitute.org/compbio1/cav.php) (I Jungreis, M Lin, and M Kellis, in prep.). Figure 4F shows CodAlignView visualization of codon alignment corresponding to *CMPK1* locus in the area of sAUG and fdAUG. The alignment is shown for representative mammalian sequences from the 100-way vertebrate alignment (Rosenbloom et al. 2015). The regions between sAUG and fdAUG contain a very large number of radical amino acid substitutions that is inconsistent with purifying selection acting on most protein-coding sequences. Nonetheless, sAUG appears to be conserved as well as its occurrence in the same frame with fdAUG, suggesting evolutionary importance of translation of this region which is further supported by the lack of stop codons in this area.

### N-terminal differences could lead to differential localization of proteoforms in the cells

The evolutionary conservation of weak sAUG context suggests that the production of two proteoforms in the chosen candidates is functional. One possibility discussed earlier is differential localization of these proteoforms. Protein N terminus often contains signal peptides, which allow proteins to be transported to particular cell compartments. Translation initiation at fdAUG may remove such signal peptides, which may affect final protein product localization in the cell.

Therefore, we decided to check whether the observed changes in relative proportions of sAUG and fdAUG products affect distribution of SNAP signal inside the cell. We reasoned that N termini could be responsible for the physiological localization of their proteins and would target SNAP reporter into corresponding location. We decided to select *LIMK1* and *ISL2* cases (sP and wt constructs in Fig. 4A) guided by information on their likely localization in the cell. LIMK1 has been shown to be involved in the remodeling of cytoskeleton (both actin- and α-tubulin-based) (Gorovoy et al. 2005) and could be colocalized with microfilaments and microtubules. ISL2 is a transcriptional factor, and we could show its nuclear localization using DNA-specific stains such as DAPI.

**Figure 5.**
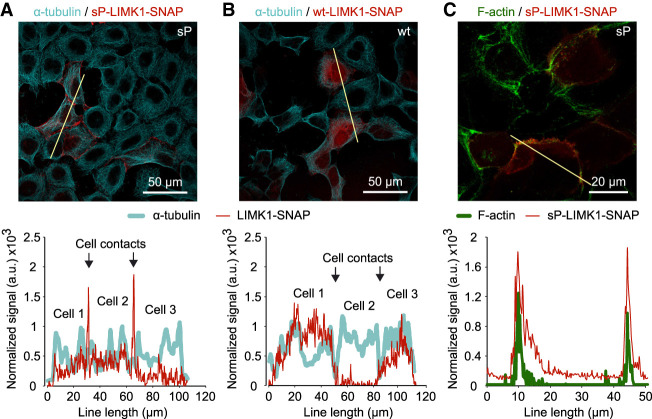
Localization of *LIMK1-SNAP*-derived products in cells. (*A*,*B*) Products from sP and wt constructs, respectively (red, stained with SNAP-Cell 647-SiR), counterstained with α-tubulin/Alexa Fluor 488 antibodies (cyan). (*Bottom*) Line profile analysis across three contacting cells shows the predominantly peripheral distribution of sP products, in contrast to more uniform cytosolic distribution of the wt products. (*C*) Colocalization of sP products (red) and F-actin (green, stained with phalloidin-Alexa 546). Images represent stacks of four (*A*,*B*) or three (*C*) focal planes taken with a 5 µm step.

We found that the products of *LIMK1* sP construct are predominantly localized in the areas of cell contacts / cell periphery, whereas products of *LIMK1* wt construct are evenly distributed in cell cytosol, with frequent, but not major increases around cell contacts (Fig. 5A,B). For both sP and wt constructs, we did not observe tight association between SNAP and microtubules. In contrast, colocalization of *LIMK1* sP construct products and F-actin was common (Fig. 5C). This suggests that the longer *LIMK1* proteoform preferentially interacts with microfilaments or F-actin-associated proteins.

The localization of ISL2-SNAP products was less affected by introducing the optimal sAUG context. However, we observed a decrease in the ratio of the nuclear and cytosolic SNAP signals upon context optimization (Supplemental Fig. S20); this is most likely attributable to a lower efficiency of longer proteoform translocation to the nucleus.

## Discussion

The scanning mechanism of translation initiation in eukaryotes and the stochastic nature of start codon selection enables plurality of start codon use. AUG codons in strong Kozak contexts are recognized very efficiently with a probability approaching 1. AUG codons in a weak context, as well as many non-AUG codons, are recognized less efficiently. This can be expressed as start codon translation initiation probabilities varying in a wide range between 0 and 1, where starts with a low probability could lead to low, yet productive levels of protein synthesis when located close to the 5′ end (Michel et al. 2014a). PICs bypassing such codons continue to scan, resulting in initiation of protein synthesis at multiple start codons on the same mRNA. Many such starts occur upstream of the regions encoding long proteins, they result in decoding of short ORFs, termed uORFs owing to their location. Although some of these uORFs encode functional microproteins and bioactive peptides, most are believed to play regulatory roles, although their dual role is also likely. Although detection and characterization of uORFs is a topic of intense research (for reviews, see Barbosa et al. 2013; Somers et al. 2013; Wethmar 2014), comparatively less attention has been paid to the plurality of start codons enabling translation of long (“main”) proteins encoded by mRNAs. We previously provided evolutionary evidence that non-AUG codons are used to produce proteoforms extended at the N terminus in comparison with AUG-initiated proteoforms in vertebrates (Ivanov et al. 2011). The existence of alternative proteoforms with non-AUG-initiated extensions with mitochondrial localization signals is also evident in yeast (Monteuuis et al. 2019). Others provided evidence for the existence of alternatively initiated proteoforms using variants of ribosome profiling (Ingolia et al. 2011; Gao et al. 2015) and mass spectrometry (Koch et al. 2014; Van Damme et al. 2014; Gawron et al. 2016). However, the functional and evolutionary significance of N-terminally truncated proteoforms was not clear.

Here, we explored the functional significance (as it applies to fitness) of such truncated proteoforms by analyzing the relationship between the contextual strengths of first AUG codons of protein-coding ORFs and the corresponding first downstream AUG codons as well as the relative phase of reading frames in which they occur. We have shown that positions as well as contexts of fdAUGs are under evolutionary selection downstream from sAUGs in a weak context in which in-frame fdAUGs are preferred over out-of-frame fdAUGs. The conservation of weak contexts at some sAUGs strongly suggests that the plurality of start codons that enables production of alternative proteoforms increases the fitness.

The experimental assessment of initiation plurality in 10 human genes with annotated start codons (sAUG) in a conserved weak context confirmed high efficiency of initiation at downstream AUG codons exceeding that of the annotated. This is supported by publicly available ribosome profiling and mass spectrometry data. In addition, our experimental data suggest that the probability of initiation at specific AUG codons is influenced by factors other than immediate nucleotide context, as in the example of *CMPK1*, in which initiation at sAUG remains minor even if placed in the perfect Kozak context. It is well known that specific mRNA elements can stimulate initiation at suboptimal codons (e.g., stem–loop structures positioned downstream from start codon) (Kozak 1990). Our observations points to the existence of alternative mRNA elements which, in contrast, can repress initiation at optimal starts. These elements may act synergistically with poor start codon context to further increase the “leakiness” of start codon.

For two selected examples (*LIMK1* and *ISL2*), we assessed how different N-termini extensions influence localization of SNAP-derived reporters and observed differences supporting the idea that proteoforms whose synthesis has initiated from different starts could be targeted to different cellular compartments. It has been shown recently that start codon selection is significantly altered in a number of stresses and pathophysiological conditions (e.g., Andreev et al. 2015; Sendoel et al. 2017). It is tempting to think that alternatively initiated proteoforms in some genes evolved in response to cellular conditions by changing localization or stability of the cumulative gene products.

In conclusion, our study showed that the plurality of translation initiation has functional significance by revealing the evolutionary selection acting on occurrence of AUG codons downstream from annotated starts. We confirmed alternative translation initiation downstream from starts in weak Kozak context using analysis of ribosome profiling data and expression reporters. The latter indicated, however, that Kozak context is not the only factor determining the leakiness of start codons; in a specific example of *CMPK1* mRNA, the optimal Kozak context is insufficient for the efficient initiation. We suspect that translation initiation at *CMPK1* sAUG may be regulated by a yet unknown regulatory mechanism because the protein sequence encoded between two AUG codons is not conserved among mammals but sAUG and its framing with fdAUG are highly conserved. Our study has direct implications to how protein-coding genes are annotated, and it reinforces the plea to incorporate information on multiple proteoforms expressed from the same mRNA molecules (Brunet et al. 2018). It is also pertinent for understanding evolution of protein-coding genes and interpretation of the effects of genomic variants in the beginnings of protein-coding genes.

## Methods

### A data set of human mRNA sequences with different Kozak contexts

A total number of 42,989 human mRNA sequences were downloaded from RefSeq database (ftp://ftp.ncbi.nlm.nih.gov/refseq/) on July 2017 (Release 83). Only manually curated mRNA sequences were used (those with accession prefix NM_). Sequences without an AUG translation initiation codon and without at least 6 nt upstream of AUG were discarded. To avoid the redundancy caused by multiple transcript variants, we took advantage of APPRIS database (Rodriguez et al. 2018) and removed isoforms not classified as principal isoforms in APPRIS RefSeq gene data set version 108. For those cases in which more than one principal isoform exists for one gene, both were used if the sequence between 6 nt upstream of sAUG and 15 nt downstream from fdAUG differed. If this sequence stretch was identical between principal isoforms, only the longest isoform was used. mRNA sequences without any AUG downstream from sAUG in the CDS were also discarded. This procedure resulted in 18,297 mRNA sequences corresponding to 18,051 genes.

Translation initiation efficiency (TIE) values were assigned to each mRNA according to previously reported data (Noderer et al. 2014), and the sequences were sorted based on these values. From this set, two equal sized subsets were created: one with 10% of the mRNAs with the highest translation initiation context efficiency values (“strong” set; 1830 mRNAs) (Supplemental Table S1) and another with the 10% of the mRNAs with the lowest translation initiation context efficiency values (“weak” set; 1830 mRNAs) (Supplemental Table S2). These data sets were then used for exploring distances between sAUGs and fdAUGs, their relative framing, and ribosome profiling densities.

### Translation initiation efficiency selection score (TIESS)

For all selected human mRNAs, sequences of orthologs were obtained from the hg38 100-way alignment (Rosenbloom et al. 2015). To measure the conservation of the context efficiency within each orthologous group, rather than conservation of the context sequence, we devised a TIESS score that has been calculated according to the following equation:

(1) $$TIESS\;=\;\overset{i}{\underset{i\;=\;1}{\sum }}\log_{10}\frac{TIE_{i}}{\bar{TIE}}\;,$$

where *i* is an orthologous sequence, which can be found in 100-way alignment; and $\bar{TIE}$ is an average TIE over all sAUG codons (all mRNAs and their orthologs from 100-way alignment).

### Ribosome profiling data analysis

For the analysis of ribosome footprint densities surrounding AUG codons, aggregated ribosome profiling data were downloaded from the GWIPS-viz browser (Michel et al. 2014b, 2018) on March 7, 2019 (for the list of ribosome profiling studies used, see Supplemental Table S5). The data represent inferred positions of A-site codons for elongating ribosomes and P-site codons for initiating ribosomes. To generate metagene profiles for the subsets of transcripts containing sAUGs in weak or strong contexts followed by in-frame or out-of-frame fdAUGs, the data were normalized for each individual transcript and median density for each codon position was represented to generate metagene profiles. The transcripts used are the same as in Supplemental Tables S1 and S2, with an exception that transcripts with a coding region shorter than 200 nt were excluded as well as transcripts with fewer than 200 aligned footprints. In addition, for the metagene profiles relative to sAUGs, transcripts with 5′ leaders shorter than 45 nt were also discarded. For the metagene profiles relative to fdAUGs, only those transcripts for which the distance between sAUG and fdAUG was >40 nt were used. This was needed to make sure that footprint density measured upstream of fdAUG does not contain footprints produced by initiating ribosomes. The footprint density surrounding AUG codons was measured for the region of −15 to −3 codons (upstream) and +10 to +44 codons (downstream). Statistical significance of the density difference was assessed with a Mann–Whitney *U* test using distributions of normalized read density merged from all genes.

### Visualization and statistical analysis

Ribosome footprint densities for individual transcripts in Figure 2 were visualized with Trips-Viz browser (Kiniry et al. 2019) using aggregated data listed in Supplemental Table S6. All other plots were generated using Python Matplotlib library (Hunter 2007) and R ggplot2 package (Wickham 2009). Statistical analyses were performed with the Statistical Functions module from SciPy library and with R (R Core Team 2013). Analysis of microscopy and immunostaining data was performed using FluoView 4.2 software (Olympus) and Microsoft Excel.

### Cloning

For each of the selected genes, the test sequence starts 30 nt upstream of the sAUG and ends 6 nt downstream from the fdAUG. For each of the candidate sequences, three controls were generated: First, the sAUG was placed in an optimal Kozak context (ACCAUGG); second, the fdAUG codon was changed to an AUC; and third, the fdAUG was placed in perfect Kozak context.

The designed sequences were synthesized by Twist Bioscience as gene fragments and then cloned upstream and in frame with the sequence encoding SNAP tag in the plasmid described below.

An insert containing SNAP tag amplified from pSNAPf (NEB) was cloned into pcDNA3.4 to generate the acceptor plasmid. The latter was further modified so that the SNAP tag initiating AUG and the next in-frame AUG codons were changed to AAG codons.

### Tissue culture and cell transfection

Human Embryonic Kidney 293T (HEK293T) cells (ATCC), Human Embryonic Kidney 293A (HEK293A) cells (ATCC), and HeLa cells (ATCC) were maintained as monolayer cultures, grown in DMEM (Sigma-Aldrich) supplemented with 10% FBS, 1 mM L-glutamine and antibiotics at 37°C in an atmosphere of 5% CO_2_. Then, 4 × 10^6^ HEK293T and HEK293A or 2 × 10^6^ HeLa cells were plated on 10-cm tissue culture dishes. After 24 h, the cells were detached with trypsin, suspended in fresh media, and transfected with Lipofectamine 2000 reagent (Invitrogen), using the one-day protocol in which suspended cells are added directly to the DNA complexes in 24-well plates. For each transfection, the following was added to each well: 200 ng plasmid DNA, 1.3 μL Lipofectamine 2000, and in 200 μL Opti-MEM (Gibco). Next, 2 × 10^5^ HEK293T or HEK293A and 1 × 10^5^ HeLa cells in 800 μL DMEM were added to the transfecting DNA complexes in each well. Transfected cells were incubated for 28 h at 37°C in 5% CO_2_.

### Protein isolation and electrophoresis

Cells were washed with 1× PBS, and whole-cell lysates were prepared in a standard RIPA buffer supplemented with 0.01 μM SNAP-Cell 647-SiR fluorescent substrate (NEB). Cells were incubated with lysis buffer for 30 min at room temperature with shaking. Lysates were then clarified by centrifugation.

Proteins were separated by 4%–12% polyacrylamide gel electrophoresis on premade BoltTM 4%–12% BisTris Plus gels (Thermo Fisher Scientific). Protein gels were scanned with Typhoon Trio+ instrument (Amersham) using the 670 BP 30 emission filter.

### Cell staining with a SNAP substrate and fixation

For immunostaining and confocal imaging, HEK293A cells were seeded at 1 × 10^4^ cells/cm^2^ on glass bottom dishes (MatTek). After 24-h incubation, plasmids encoding LIMK1-SNAP and ISL2-SNAP (P and WT) were delivered in cells by incubating them with DNA complexes in Opti-MEM for 4 h; 100 ng DNA and 0.2 μL Lipofectamine 2000 were used per 1 cm^2^. Then, cells were grown in standard DMEM supplemented with 10% FBS for 14 h before staining with SNAP-Cell 647-SiR (NEB) conducted in DMEM in CO_2_ incubator (2 μM for 30 min). Cells were washed with prewarmed DMEM (two times) and Dulbecco PBS with Ca^2+^ and Mg^2+^ (two times), and then fixed with either 4% paraformaldehyde (PFA, 10 min, for phalloidin staining) or cold 100% methanol (15 min at −20°C for α-tubulin staining).

### Immunostaining and confocal microscopy

Cells were permeabilized with 0.25% (PFA-fixed) or 0.1% TX100 (methanol-fixed) and blocked with 5% FBS in standard TBST (0.1% Tween-20 at pH 7.6). To visualize microtubules, methanol-fixed cells were incubated with primary α-tubulin (Sigma-Aldrich T5168, 1:500 dilution) and secondary Alexa Fluor 488-conjugated (Thermo Fisher Scientific A21200, 1:1000 dilution) antibodies for 1 h in blocking solution. TBST was used to wash cells (5 × 5 min). To visualize F-actin, PFA-fixed cells were stained with Alexa Fluor 546 phalloidin (5 units/mL for 20 min in 1% BSA in PBS) and DAPI (2 μM for 20 min in PBS).

Cells were kept in PBS for confocal imaging. Fluorescence and differential interference contrast (DIC) images were collected on an Olympus FV1000 confocal laser-scanning microscope using oil immersion UPLSAPO 60×/1.35 Super Apochromat objective. DAPI, Alexa Fluor 488, Alexa Fluor 546, and SNAP-Cell 647-SiR dyes were excited with 405, 488, 543, and 633 nm lasers, respectively; separate fluorescence signals were collected with 0.5 μm steps in sequential laser mode with emission gates adjusted to avoid spectral overlap.

All sequences and data used in this work are publicly available. Accession IDs for all RefSeq sequences used are provided in Supplemental Tables S1–S4, and IDs for all raw sequencing data are provided in Supplemental Tables S5 and S6.

## Competing interest statement

The authors declare no competing interests.

## Supplementary Material

Supplemental Material
