# Supplementary material for: Translation initiation downstream from annotated start codons in human mRNAs coevolves with the Kozak context
Source: Genome Res. 2020 Jul;30(7):974–84. doi: 10.1101/gr.257352.119 (PMC7397870; doi:10.1101/gr.257352.119)
Supplement: Supplemental Material [file supp_30_7_974__index.html]

Translation initiation downstream from annotated start codons in human mRNAs coevolves with the Kozak context — Translation initiation downstream from annotated start codons in human mRNAs coevolves with the Kozak context — Supplemental Material 

# Translation initiation downstream from annotated start codons in human mRNAs coevolves with the Kozak context

## Supplemental Material

- Supplemental\_Material.pdf
- Supplemental\_Table\_S1.xlsx
- Supplemental\_Table\_S2.xlsx
- Supplemental\_Table\_S3.xlsx
- Supplemental\_Table\_S4.xlsx
- Supplemental\_Table\_S5.xlsx
- Supplemental\_Table\_S6.xlsx
