# Supplementary material for: Translation initiation downstream from annotated start codons in human mRNAs coevolves with the Kozak context
Source: Genome Res. 2020 Jul;30(7):974–84. doi: 10.1101/gr.257352.119 (PMC7397870; doi:10.1101/gr.257352.119)
Supplement: Supplemental Material [file supp_gr.257352.119_Supplemental_Material.pdf]

Supplemental Material accompanying

Translation initiation downstream of annotated start codons in human mRNAs coevolves with the Kozak context.

by

Maria S. Benitez-Cantos, Martina M. Yordanova, Patrick B.F. O'Connor, Alexander V. Zhdanov, Sergey I. Kovalchuk, Dmitri B. Papkovsky, Dmitry E. Andreev & Pavel V. Baranov

Supplemental Figures

Supplemental Figure S1. Distribution of ORF lengths initiated at fdAUGs.

Supplemental Figure S2. Further experimental verification of downstream initiation at mRNAs containing sAUGs in evolutionary conserved weak context.

Supplemental Figure S3. GWIPS-viz screenshot of *AASDHPPT* locus of the reference human genome (hg38 assembly) in the vicinity of sAUG and fdAUG.

Supplemental Figure S4. GWIPS-viz screenshot of *ASPHD1* locus of the reference human genome (hg38 assembly) in the vicinity of sAUG and fdAUG.

Supplemental Figure S5. GWIPS-viz screenshot of *C1orf94* locus of the reference human genome (hg38 assembly) in the vicinity of sAUG and fdAUG.

Supplemental Figure S6. GWIPS-viz screenshot of *CMPK1* locus of the reference human genome (hg38 assembly) in the vicinity of sAUG and fdAUG.

Supplemental Figure S7. GWIPS-viz screenshot of *FRMD3* locus of the reference human genome (hg38 assembly) in the vicinity of sAUG and fdAUG.

Supplemental Figure S8. GWIPS-viz screenshot of *ISL2* locus of the reference human genome (hg38 assembly) in the vicinity of sAUG and fdAUG.

Supplemental Figure S9. GWIPS-viz screenshot of *LIMK1* locus of the reference human genome (hg38 assembly) in the vicinity of sAUG and fdAUG.

Supplemental Figure S10. GWIPS-viz screenshot of *PABPCL4L* locus of the reference human genome (hg38 assembly) in the vicinity of sAUG and fdAUG.

Supplemental Figure S11. GWIPS-viz screenshot of *RELB* locus of the reference human genome (hg38 assembly) in the vicinity of sAUG and fdAUG.

Supplemental Figure S12. GWIPS-viz screenshot of *ZBTB8OS* locus of the reference human genome (hg38 assembly) in the vicinity of sAUG and fdAUG.

Supplemental Figure S13. Peptide observation frequency in PeptideAtlas for *AASDHPPT* protein products.

Supplemental Figure S14. Peptide observation frequency in PeptideAtlas for *ASPHD1* protein products.

Supplemental Figure S15. Peptide observation frequency in PeptideAtlas for *FRMD3* protein products.

Supplemental Figure S16. Peptide observation frequency in PeptideAtlas for *ISL2* protein products.

Supplemental Figure S17. Peptide observation frequency in PeptideAtlas for *LIMK3* protein products.

Supplemental Figure S18. Peptide observation frequency in PeptideAtlas for *RELB* protein products.

Supplemental Figure S19. Peptide observation frequency in PeptideAtlas for *ZBTB8OS* protein products.

Supplemental Figure S20. Localization of *ISL2-SNAP* derived products in cells.

Supplemental Tables (Excel files)

Supplemental Table S1. “Strong” set.

Supplemental Table S2. “Weak” set.

Supplemental Table S3. Conserved “strong” set.

Supplemental Table S4. Conserved “weak” set.

Supplemental Table S5. Studies used in the ribosome profiling analysis.

Supplemental Table S6. Studies used for ribosome footprint density visualization.

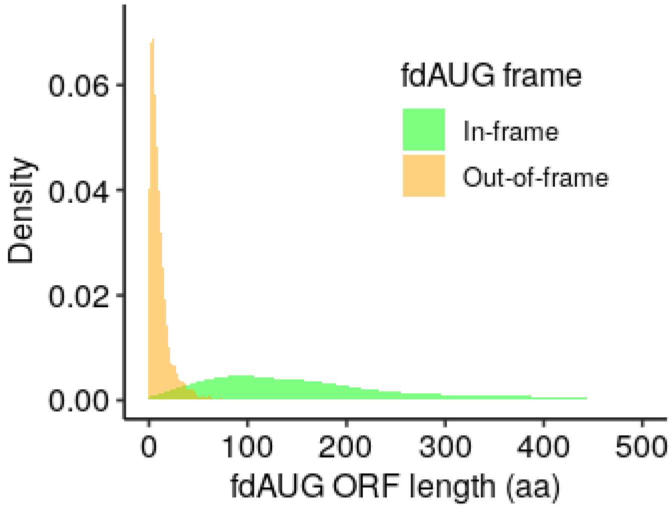

Supplemental Figure S1 I Distribution of ORF lengths initiated at fdAUGs. ORFs initiated at out-of-frame fdAUGs are significantly shorter than those initiated at in-frame fdAUGs suggesting the lack of negative evolutionary selection acting on stop codon occurrence downstream of fdAUGs.

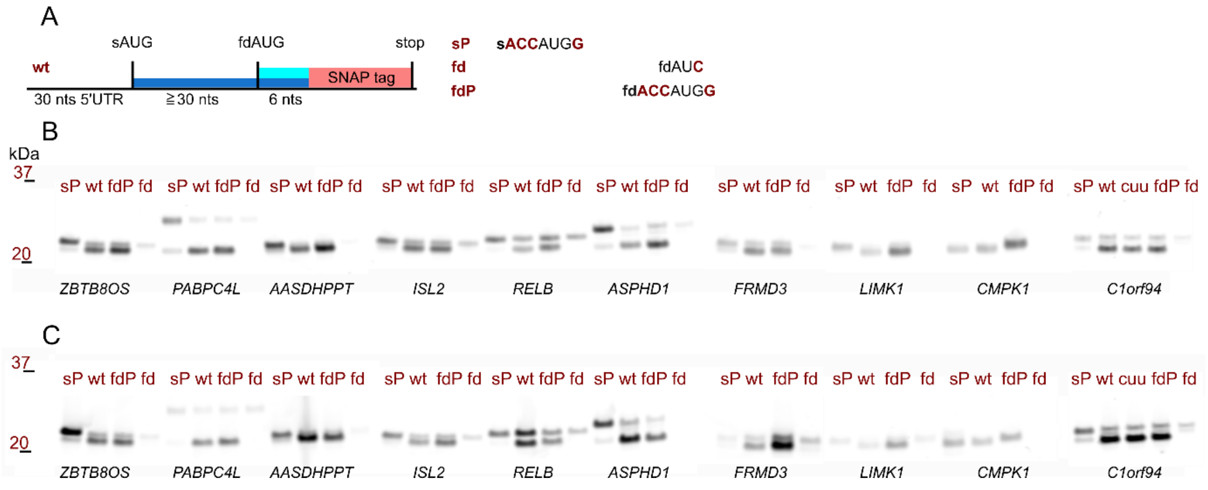

Supplemental Figure S2 | Further experimental verification of downstream initiation at mRNAs containing sAUGs in evolutionary conserved weak context. A. Schematic of the test sequence cassette fused to SNAP tag. The **wt** test sequence includes 30 nt upstream of sAUG and 6 nt downstream of fdAUG. s**P** has the sAUG in perfect Kozak context, **fd** has the fdAUG changed to AUC and fdP has the fdAUG in perfect Kozak context. **wt**, **sP**, **fd** and **fdP** test cassettes were designed for each of the 10 selected genes. **B.** Scans of protein gels used to separate SNAP-tagged protein products of the test constructs expressed in HeLa cells. **C.** Scans of protein gels used to separate SNAP-tagged protein products of the test constructs expressed in HEK293A cells. Gene names are shown below the lanes. For *C1orf94* loci in **B** and **C** there is an additional construct **cuu** in which a CUG codon between sAUG and fdAUG was changed to a CUU codon.

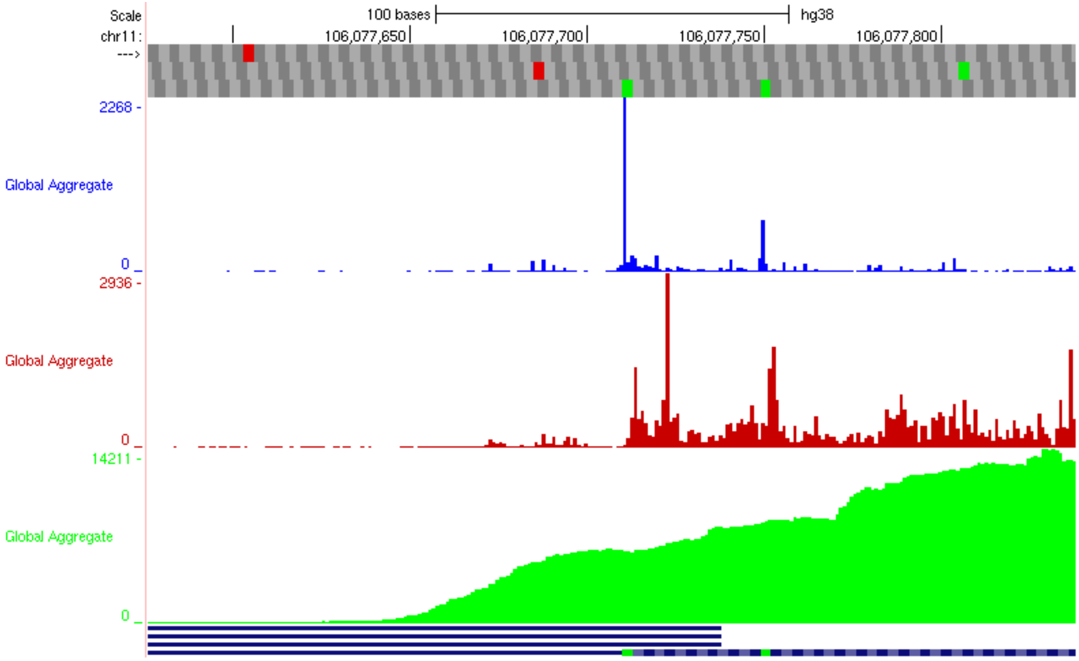

Supplemental Figure S3 I GWIPS-viz screenshot of *AASDHPPT* locus of the reference human genome (hg38 assembly) in the vicinity of sAUG and fdAUG. Tracks of aggregated ribosome profiling data are shown as the following. Blue are the locations of ribosome P-sites from ribosome footprints enriched at sites of initiation. Dark red are the locations of A-sites inferred from footprints of arrested elongating ribosomes. Refseq annotations tracks are shown at the bottom and ORF plots are at the top.

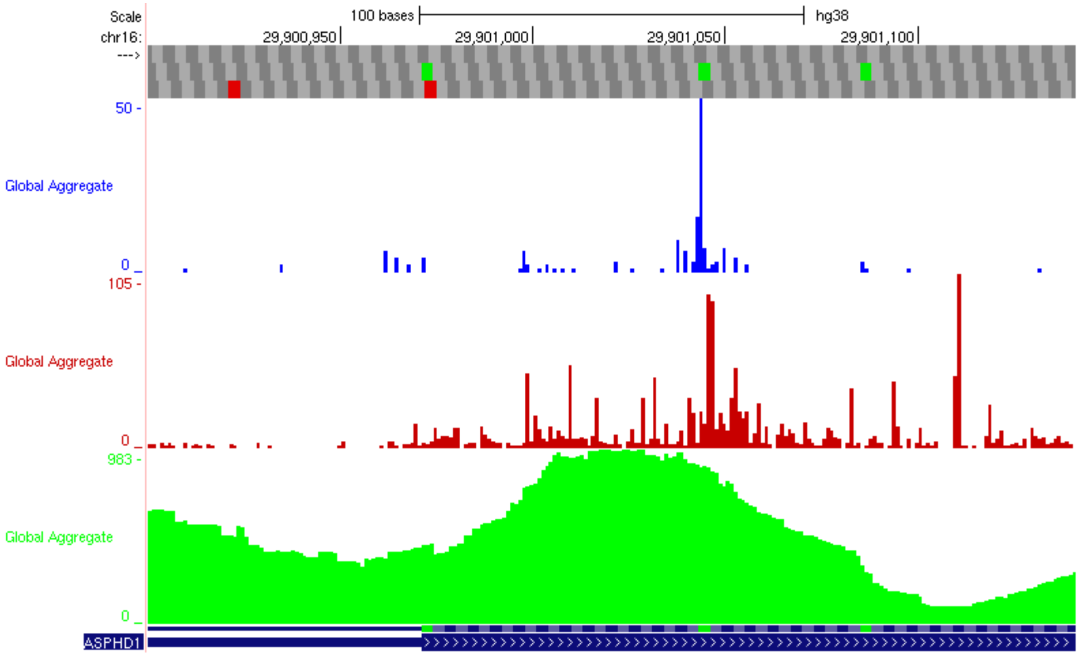

Supplemental Figure S4 I GWIPS-viz screenshot of *ASPHD1* locus of the reference human genome (hg38 assembly) in the vicinity of sAUG and fdAUG. Tracks of aggregated ribosome profiling data are shown as the following. Blue are the locations of ribosome P-sites from ribosome footprints enriched at sites of initiation. Dark red are the locations of A-sites inferred from footprints of arrested elongating ribosomes. Refseq annotations tracks are shown at the bottom and ORF plots are at the top.

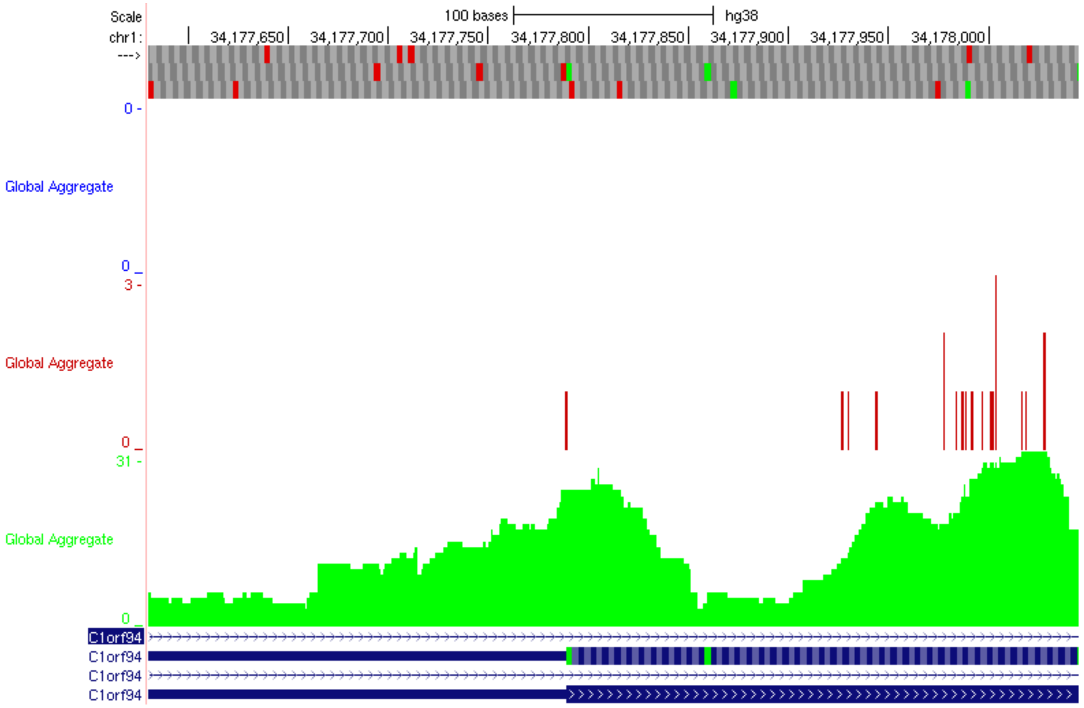

Supplemental Figure S5 I GWIPS-viz screenshot of *C1orf94* locus of the reference human genome (hg38 assembly) in the vicinity of sAUG and fdAUG. Tracks of aggregated ribosome profiling data are shown as the following. Blue are the locations of ribosome P-sites from ribosome footprints enriched at sites of initiation. Dark red are the locations of A-sites inferred from footprints of arrested elongating ribosomes. Refseq annotations tracks are shown at the bottom and ORF plots are at the top.

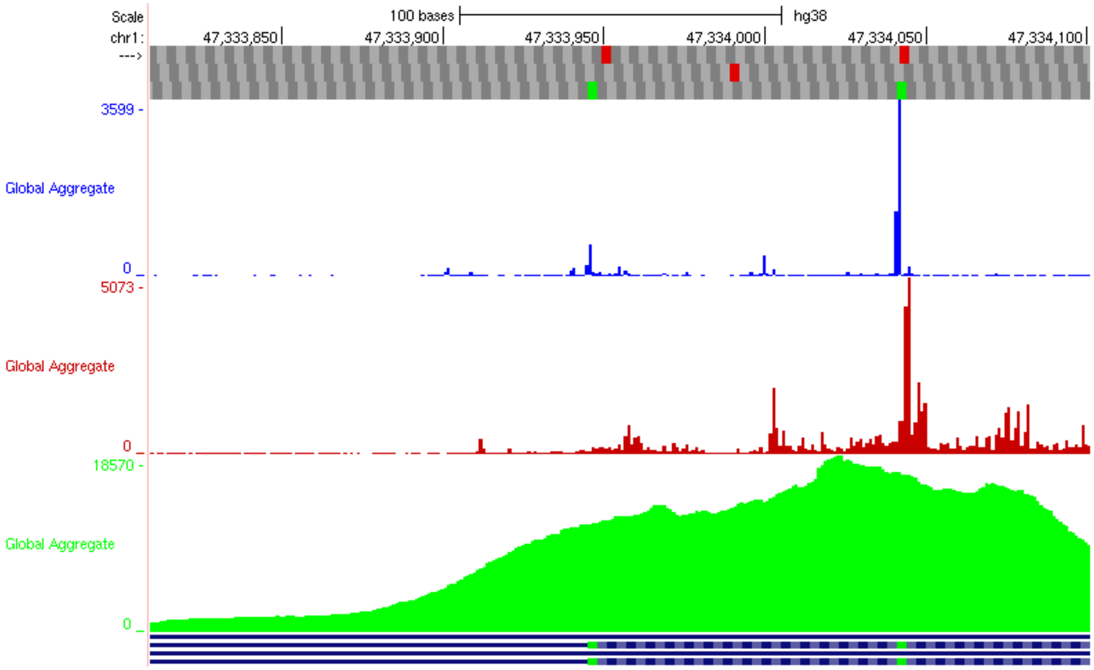

Supplemental Figure S6 I GWIPS-viz screenshot of *CMPK1* locus of the reference human genome (hg38 assembly) in the vicinity of sAUG and fdAUG. Tracks of aggregated ribosome profiling data are shown as the following. Blue are the locations of ribosome P-sites from ribosome footprints enriched at sites of initiation. Dark red are the locations of A-sites inferred from footprints of arrested elongating ribosomes. Refseq annotations tracks are shown at the bottom and ORF plots are at the top.

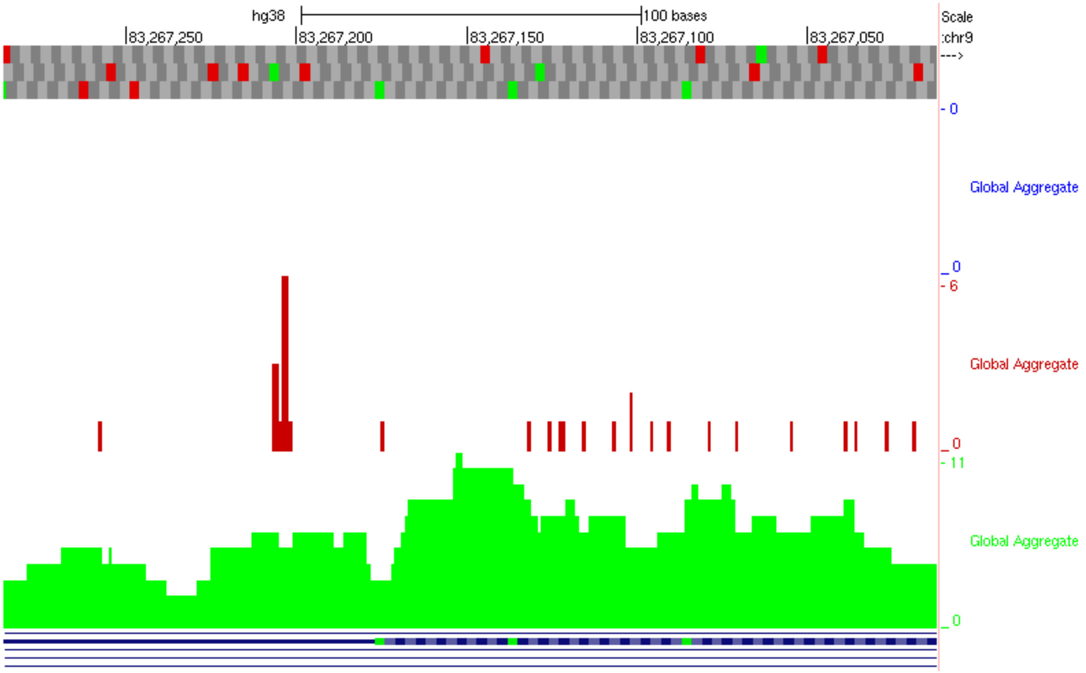

Supplemental Figure S7 I GWIPS-viz screenshot of *FRMD3* locus of the reference human genome (hg38 assembly) in the vicinity of sAUG and fdAUG. Tracks of aggregated ribosome profiling data are shown as the following. Blue are the locations of ribosome P-sites from ribosome footprints enriched at sites of initiation. Dark red are the locations of A-sites inferred from footprints of arrested elongating ribosomes. Refseq annotations tracks are shown at the bottom and ORF plots are at the top.

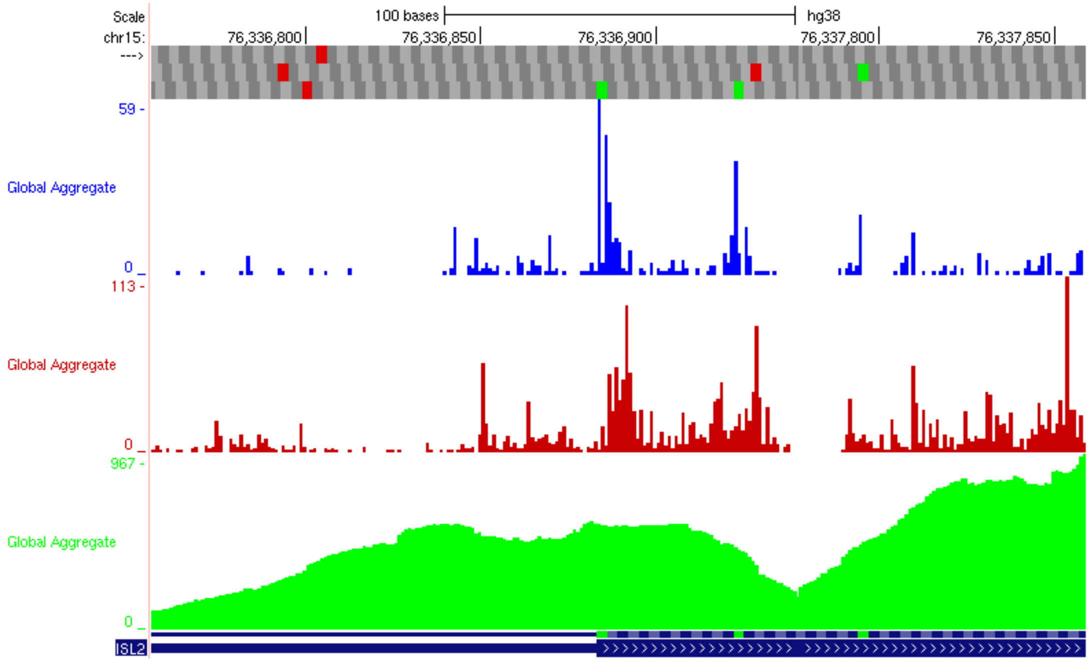

Supplemental Figure S8 I GWIPS-viz screenshot of *ISL2* locus of the reference human genome (hg38 assembly) in the vicinity of sAUG and fdAUG. Tracks of aggregated ribosome profiling data are shown as the following. Blue are the locations of ribosome P-sites from ribosome footprints enriched at sites of initiation. Dark red are the locations of A-sites inferred from footprints of arrested elongating ribosomes. Refseq annotations tracks are shown at the bottom and ORF plots are at the top.

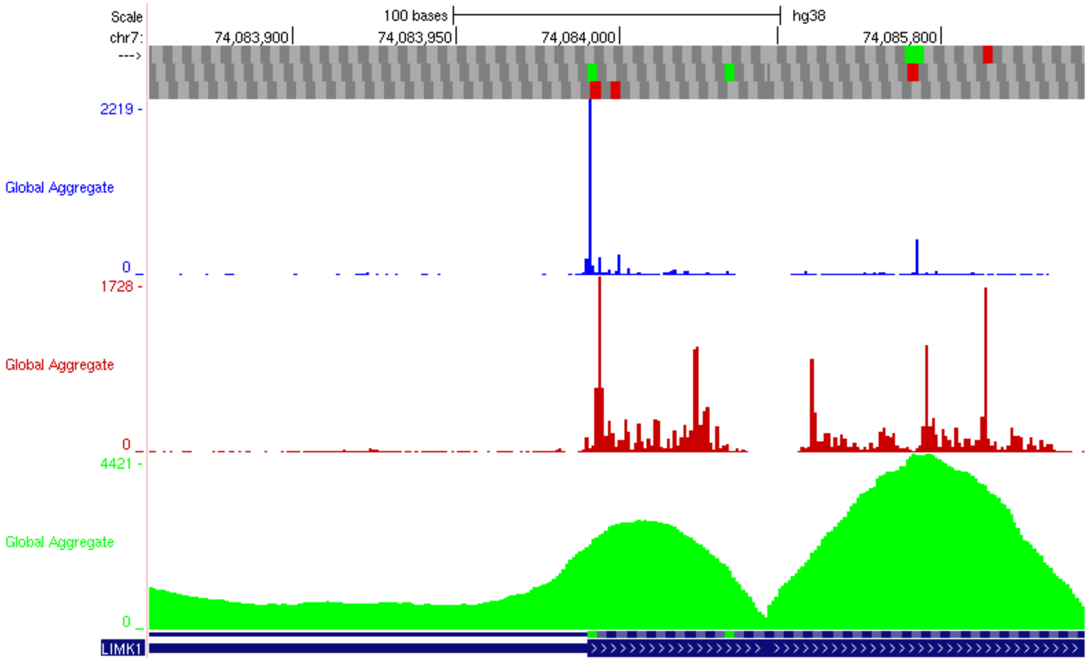

Supplemental Figure S9 I GWIPS-viz screenshot of *LIMK1* locus of the reference human genome (hg38 assembly) in the vicinity of sAUG and fdAUG. Tracks of aggregated ribosome profiling data are shown as the following. Blue are the locations of ribosome P-sites from ribosome footprints enriched at sites of initiation. Dark red are the locations of A-sites inferred from footprints of arrested elongating ribosomes. Refseq annotations tracks are shown at the bottom and ORF plots are at the top.

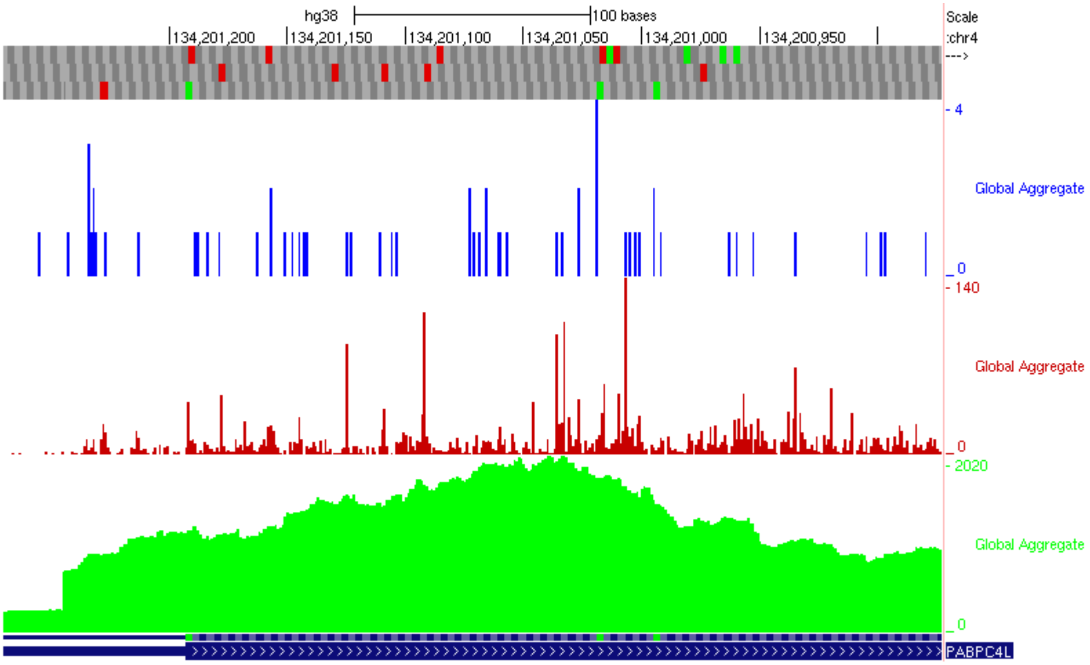

Supplemental Figure S10 I GWIPS-viz screenshot of *PABPCL4L* locus of the reference human genome (hg38 assembly) in the vicinity of sAUG and fdAUG. Tracks of aggregated ribosome profiling data are shown as the following. Blue are the locations of ribosome P-sites from ribosome footprints enriched at sites of initiation. Dark red are the locations of A-sites inferred from footprints of arrested elongating ribosomes. Refseq annotations tracks are shown at the bottom and ORF plots are at the top.

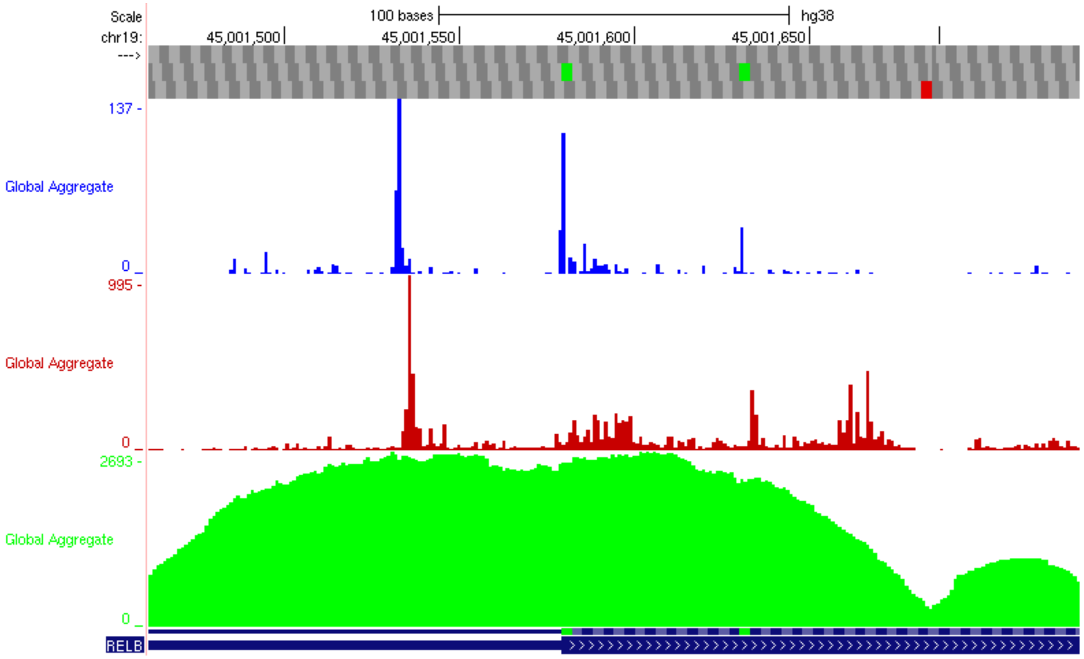

Supplemental Figure S11 I GWIPS-viz screenshot of *RELB* locus of the reference human genome (hg38 assembly) in the vicinity of sAUG and fdAUG. Tracks of aggregated ribosome profiling data are shown as the following. Blue are the locations of ribosome P-sites from ribosome footprints enriched at sites of initiation. Dark red are the locations of A-sites inferred from footprints of arrested elongating ribosomes. Refseq annotations tracks are shown at the bottom and ORF plots are at the top.

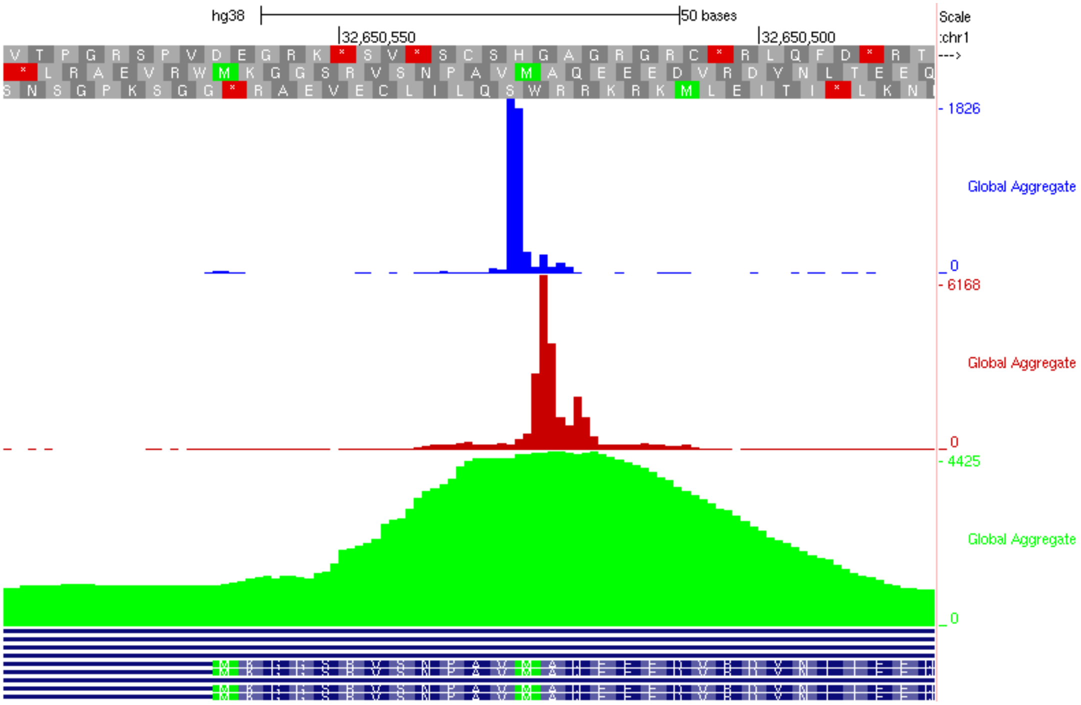

Supplemental Figure S12 I GWIPS-viz screenshot of *ZBTB8OS* locus of the reference human genome (hg38 assembly) in the vicinity of sAUG and fdAUG. Tracks of aggregated ribosome profiling data are shown as the following. Blue are the locations of ribosome P-sites from ribosome footprints enriched at sites of initiation. Dark red are the locations of A-sites inferred from footprints of arrested elongating ribosomes. Refseq annotations tracks are shown at the bottom and ORF plots are at the top.

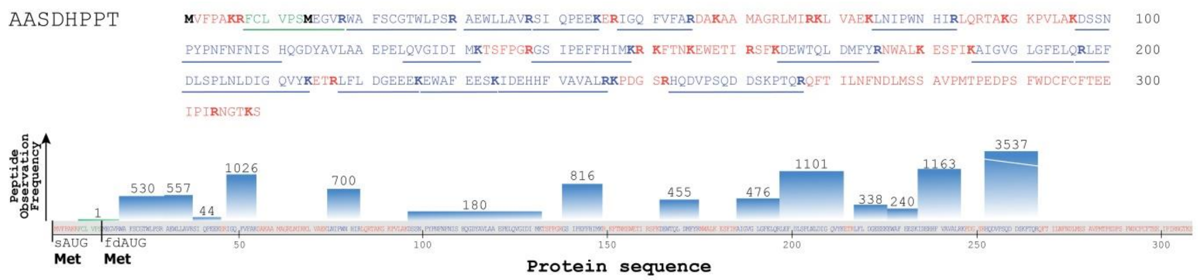

Supplemental Figure S13 I Peptide observation frequency in PeptideAtlas for *AASDHPPT* protein products. In red are the tryptic peptides not expected to be detectable. In green are detectable peptides unique for the long proteoform and in blue are shared peptides.

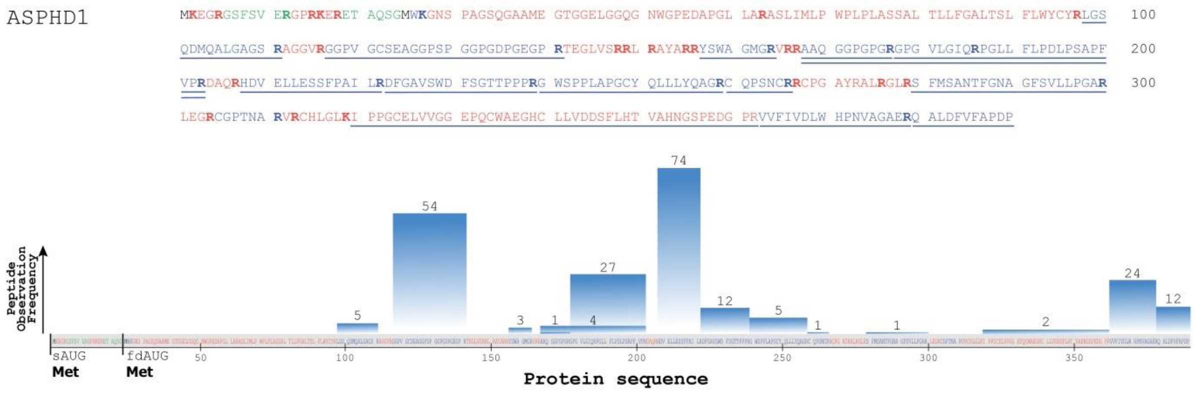

Supplemental Figure S14 I Peptide observation frequency in PeptideAtlas for *ASPHD1* protein products. In red are the tryptic peptides not expected to be detectable. In green are detectable peptides unique for the long proteoform and in blue are shared peptides.

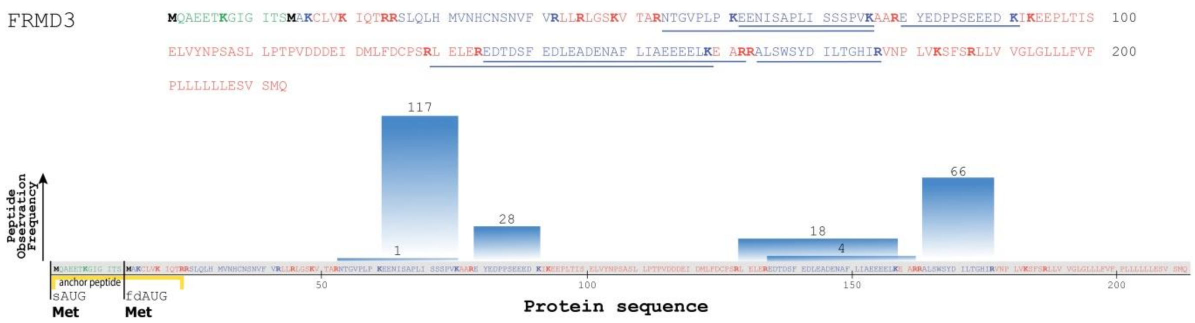

Supplemental Figure S15 I Peptide observation frequency in PeptideAtlas for *FRMD3* protein products. In red are the tryptic peptides not expected to be detectable. In green are detectable peptides unique for the long proteoform and in blue are shared peptides. Anchor peptides predicted by Signal P is marked in orange.

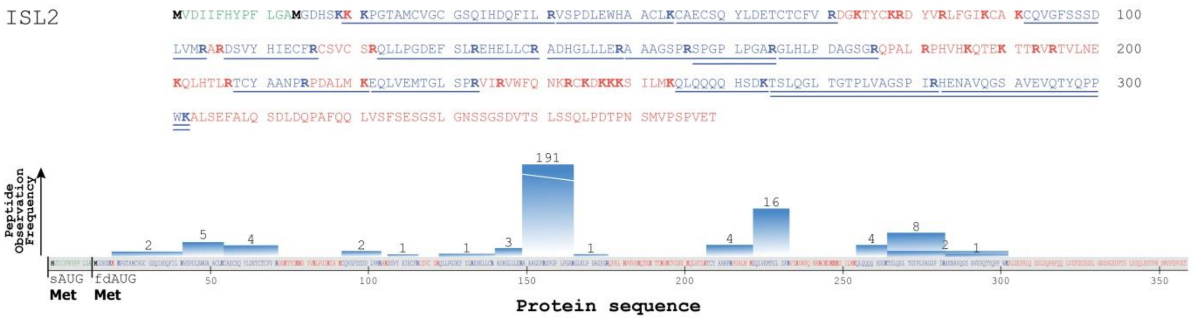

Supplemental Figure S16 I Peptide observation frequency in PeptideAtlas for *ISL2* protein products. In red are the tryptic peptides not expected to be detectable. In green are detectable peptides unique for the long proteoform and in blue are shared peptides.

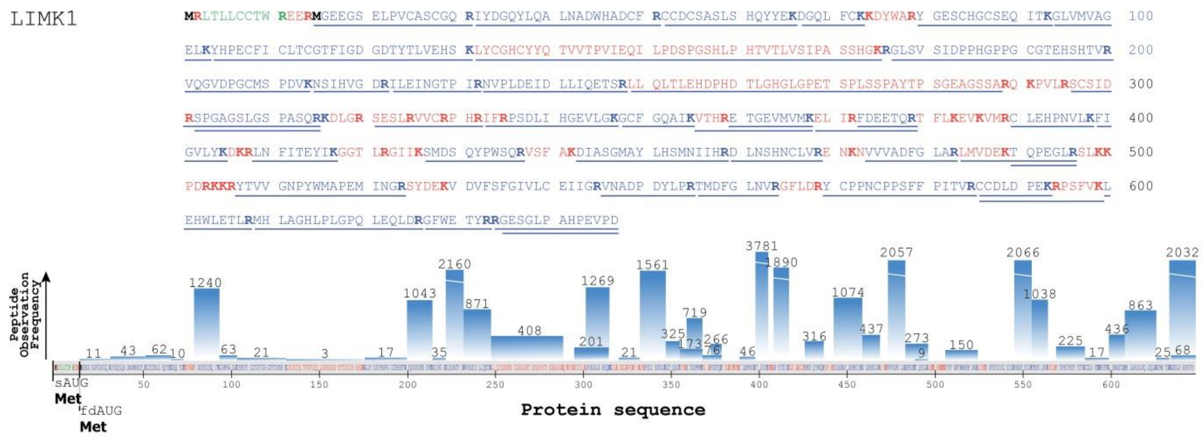

Supplemental Figure S17 I Peptide observation frequency in PeptideAtlas for *LIMK3* protein products. In red are the tryptic peptides not expected to be detectable. In green are detectable peptides unique for the long proteoform and in blue are shared peptides.

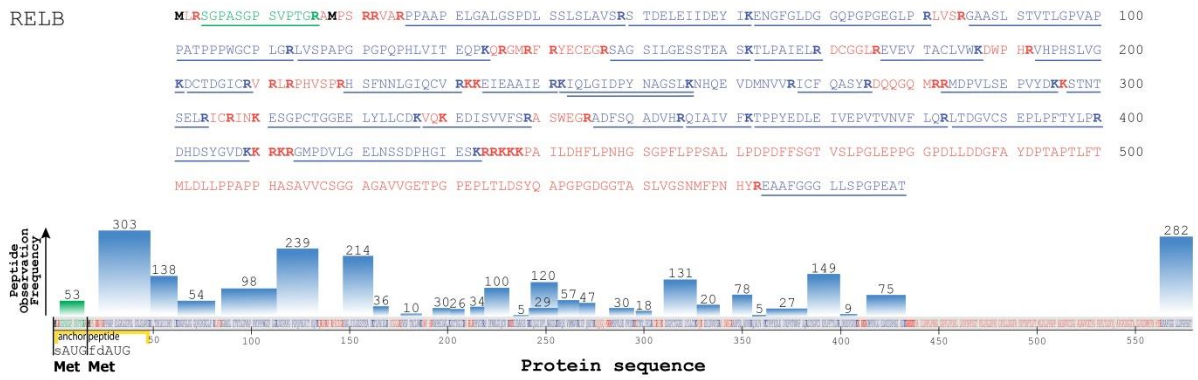

Supplemental Figure S18 I Peptide observation frequency in PeptideAtlas for *RELB* protein products. In red are the tryptic peptides not expected to be detectable. In green are detectable peptides unique for the long proteoform and in blue are shared peptides.

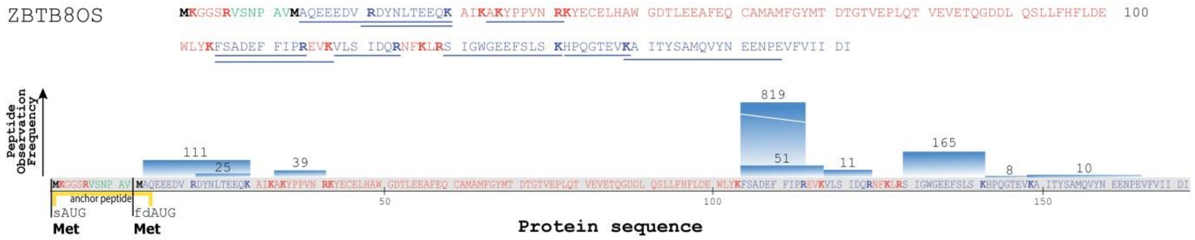

Supplemental Figure S19 I Peptide observation frequency in PeptideAtlas for *ZBTB8OS* protein products. In red are the tryptic peptides not expected to be detectable. In green are detectable peptides unique for the long proteoform and in blue are shared peptides. Anchor peptide predicted by Signal P is marked in orange.

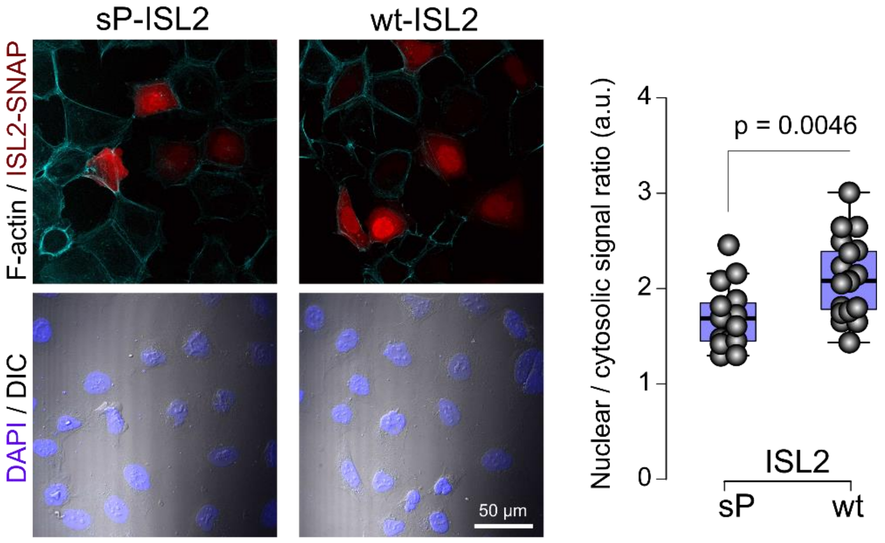

Supplemental Figure S20 I Localization of *ISL2-SNAP* derived products in cells. ISL2-SNAP (red, stained with SNAP-Cell® 647-SiR) is counterstained with Alexa Fluor 546 phalloidin (cyan) and DAPI (blue). Images represent a single plane (DIC) and stacks of five focal planes taken with a 0.5 µm step (fluorescence). In the right panel, relative proportion of ISL2 P-SNAP (N = 16) and ISL2 WT-SNAP (N = 19) products localized to the nuclei was cross-compared using *t*-test.
